# Risperidone-Induced Urinary Incontinence in Schizoaffective Disorder: A Case Report

**DOI:** 10.7759/cureus.68121

**Published:** 2024-08-29

**Authors:** Bashayer M Almaazmi, Pearl Rex, Abdullah N Aldweik, Mazin A Mukhtar

**Affiliations:** 1 Psychiatry, Al Amal Psychiatric Hospital, Dubai, ARE; 2 Medicine, University of Sharjah, Sharjah, ARE

**Keywords:** psychopharmacology, medication side-effects, antipsychotics, urinary incontinence, risperidone

## Abstract

Risperidone is a widely used atypical antipsychotic known for its efficacy in managing various psychiatric conditions. However, it is not without adverse effects, and one such underreported side effect is that of urinary incontinence. This case report highlights the experience of a 59-year-old gentleman with a diagnosis of schizoaffective disorder who, after being admitted due to a relapse of his symptoms, developed urinary incontinence with risperidone. The patient's symptoms resolved upon the gradual reduction of the risperidone dose to 2 mg at bedtime.

Urinary incontinence as a side effect of antipsychotics has been documented in children, especially in those with autism or developmental disorders, but there is limited research on its occurrence in adults. Urinary incontinence can have significant social and psychological impacts on patients, leading to feelings of embarrassment and social withdrawal. It can also contribute to treatment non-adherence leading to frequent relapses and exacerbations of psychiatric symptoms.

In managing patients with risperidone-induced urinary incontinence, it is essential to explore medical causes thoroughly before considering medication changes. Behavioral modifications such as bladder training, biofeedback, and lifestyle modifications can also be effective in reducing the frequency of incontinence episodes. This case report emphasizes the importance of prompt identification and intervention to minimize the debilitating effects of urinary incontinence on patients with psychiatric conditions.

## Introduction

Risperidone is a second-generation antipsychotic medication. It is indicated for use in a variety of psychiatric conditions, and these conditions include but are not limited to schizophrenia, bipolar disorder, delusional disorders, and autism-associated irritability [1]. This drug induces its effects primarily via antagonism of dopamine (D2) and serotonin receptors (5HT2A), and it also interacts with other receptors such as alpha-adrenergic receptors and histamine receptors [2]. Despite its varied uses, risperidone is not without adverse effects, with some of them being more debilitating than others. As a second-generation anti-psychotic, its antagonistic effect on D2 receptors is less pronounced than that of first-generation antipsychotics, which is why the extrapyramidal symptoms that risperidone could evoke such as akathisia, dyskinesia, and dystonia are not that prominent. However, dopamine leads to the inhibition of prolactin, and thus antagonism of D2 receptors in the tuberoinfundibular pathway leads to hyperprolactinemia. This can cause sexual dysfunction, gynecomastia, and galactorrhea in males, and galactorrhea and amenorrhea in females. Other metabolic changes such as dyslipidemia, hyperglycemia, and weight gain have also been observed [3]. One underreported but rather debilitating side-effect of risperidone is urinary incontinence [4], i.e., the unintentional passing of urine, which is what the patient in this case report had endured. This side effect could be socially isolating for the patient, which is an undesirable outcome given that it could contribute to the deterioration of the well-being of the afflicted. Additionally, it could hamper patient compliance, and as a result, it would affect the management and progression of the patient’s disorder. To minimize these consequences, prompt identification and intervention are necessary.

## Case presentation

This is the case of a 59-year-old Syrian gentleman who was brought by his wife to our emergency department due to a two-week history of persecutory delusions, an elated mood, restlessness, and overspending. This is on the background of a 20-year history of schizophrenia and a one-month history of medication noncompliance.

Our patient was diagnosed with schizophrenia in 1998 and was reported to be stable on Zuclopenthixol 100 mg IM, administered every two weeks. However, due to the war-torn conditions of his home country of Syria, our patient immigrated and, as a result, was lost to his usual follow-ups. He also missed a month of medications.

Collateral history from our patient’s wife highlighted a two-week history of paranoid and persecutory delusions pertaining to his family members, the content of which included beliefs that his family members are possessed by evil spirits as well as partaking in illicit drug use. The patient also believed that he was being tracked by various governmental bodies. This escalated when he began threatening to burn his house and, soon enough, began switching on the gas and stove in the kitchen. He also tried to injure his wife and daughter with lit cigarettes. He was also noted to be extremely irritable, easily provoked, and recklessly overspending when it came to his finances.

Of note, he had no past medical history, no known allergies, and no history of substance misuse.

He was admitted to an acute psychiatric ward after medical clearance. Over the course of his inpatient interviews, in addition to his persecutory delusions, the team noted an increase in sexual-oriented topics, elated mood, inflated self-esteem, increased energy levels despite a lack of sleep, talkativeness, and grandiose delusions. As a result, under the diagnostic criteria of the ICD-10, a diagnosis of schizoaffective disorder was placed.

He was started on risperidone at 2 mg PO bedtime, with the dose gradually increasing to 6 mg PO bedtime over the course of three weeks. Five days later, the patient began to complain of urinary incontinence, stating that it interrupted his daily prayers as a practicing Muslim and interrupted his sleep, occurring more than six times at night. The medical workup was unremarkable. Upon gradual reduction of the dose of Risperidone to 2 mg PO Bedtime, his incontinence ceased.

Our patient was stabilized on a regimen of risperidone 2 mg PO bedtime, sodium valproate 500 mg PO BID, and long-acting paliperidone 100 mg IM every four weeks.

## Discussion

The aim of this report is to draw attention to the frequently neglected yet noteworthy side effects of the atypical antipsychotic risperidone. The preexisting documented cases in the literature of urinary incontinence related to risperidone use have been limited to children, particularly those with autism or other developmental conditions, with the few documented cases predominantly in the United States. To our knowledge, this would be the first reported case of risperidone-induced urinary incontinence in an adult in the Middle Eastern region.

Urinary incontinence as a side effect of antipsychotics, as per studies, has an incidence of less than 0.23-30% and a prevalence of 1-42% [5]. Incontinence resulting from antipsychotic use is typically considered when the issue arises after the drug has been prescribed and often resolves upon discontinuation of the medication.

A cohort study done in New Zealand concluded that the incidence rate of nocturnal enuresis in patients taking atypical antipsychotics was 20% for clozapine, 9.6% for olanzapine, 6.7% for quetiapine, and 6.2% for risperidone [6].

Despite its importance, limited research has focused on the exact mechanism responsible for this side effect. In the case of risperidone, alpha-1-adrenergic blockade has been proposed as a potential mechanism for urinary incontinence due to its function of regulating the internal bladder sphincter [7]. Risperidone is metabolized to its active form primarily by the enzyme CYP2D6, with a lesser contribution from CYP3A4. Patients who are poor metabolizers of risperidone, characterized by having two inactive copies of the CYP2D6 gene, tend to accumulate risperidone instead of its active metabolite [8]. While these patients may experience larger reductions in symptoms of schizophrenia, they are also more prone to extrapyramidal symptoms and potentially enuresis. Therefore, patients who exhibit significant improvement in psychotic symptoms on risperidone but develop enuresis may lack functional CYP2D6 enzymes, resulting in an increased blockade of alpha-1-adrenergic receptors.

Its active form, 9-hydroxyrisperidone, can be directly taken via paliperidone, which has weaker effects on these receptors compared to risperidone [9].

Urinary incontinence can also have a significant impact on both patients and caregivers. Adding to the stigma of the preexisting psychiatric illness, the side effect of incontinence could be a factor leading to episodes of indignity and humiliation. For patients who are dependent on caregivers, managing hygiene frequently becomes a challenge, which can eventually increase the risk of caregiver fatigue and burnout.

The episodes can also prompt patients to be more socially withdrawn to avoid further instances of incontinence in outdoor settings. Some patients might refrain from reporting this issue, possibly due to feeling embarrassed about discussing it with medical staff. Clinicians should also keep in mind urinary incontinence as a causative factor for relapses, as it can by itself lead to non-adherence to treatment [10].

In psychiatry, prioritizing the prevention of incontinence and providing timely treatment can significantly ease the journey of patients through their illness. Doing so not only reduces feelings of embarrassment but also boosts self-confidence and enhances social acceptance.

When a patient presents with new-onset urinary incontinence, it is imperative to explore medical causes thoroughly before considering medication changes. Recommended practice highlights the need to exclude common causes of urinary incontinence, such as infections caused by gonorrhea, chlamydia, acute cystitis, diabetes mellitus, or, in males, prostatic enlargement. Medication interactions, as well as the consideration of over-the-counter drugs, should be explored as well [11]. When considering a decrease in the dose of the antipsychotic to a medication that does not cause urinary incontinence or that decreases its frequency to a level that no longer distresses the patient, it is important to ensure that psychiatric complaints are controlled under the new medication or dose. If the patient is stable under the current medication, a conversation outlining the risk of medication transition, with attention given to the deterioration of mental state, should be held between the psychiatrist and the patient.

Some case studies highlight medication management for patients unable to tolerate a decrease in dose or a change in medication. Medications that have been used include oxybutynin, pseudoephedrine, mirabegron, imipramine, and duloxetine, as well as Botox injections [12].

However, it is recommended that providers educate patients on behavioral modifications to reduce the frequency of the incontinence episodes, such as bladder training, biofeedback, pelvis muscle exercises, timed and/or prompted voiding, lifestyle modifications that are inclusive of fluid management, caffeine reduction, and symptom relief [13].

## Conclusions

A 59-year-old gentleman diagnosed with schizoaffective disorder developed urinary incontinence after being prescribed risperidone. The patient's symptoms resolved upon the gradual reduction of the risperidone dose to 2 mg at bedtime. This case report highlights the underreported side effects of risperidone-induced urinary incontinence, particularly in adults. Prompt identification and intervention are crucial to minimize the debilitating social and psychological effects of urinary incontinence on patients with psychiatric conditions.
